# Implementation of the Identification and Referral to Improve Safety programme for patients with experience of domestic violence and abuse: A theory‐based mixed‐method process evaluation

**DOI:** 10.1111/hsc.12733

**Published:** 2019-03-13

**Authors:** Natalia V. Lewis, Anna Dowrick, Alex Sohal, Gene Feder, Chris Griffiths

**Affiliations:** ^1^ Centre for Primary Care and Public Health Queen Mary University of London London UK; ^2^ Centre for Academic Primary Care, Bristol Medical School, University of Bristol Bristol UK

**Keywords:** domestic violence, general practice, implementation research, primary health and social care interface, primary healthcare, process evaluation

## Abstract

**I**dentification and **R**eferral to **I**mprove **S**afety (IRIS) is a training and support programme to improve the response to domestic violence and abuse (DVA) in general practice. Following a pragmatic cluster‐randomised trial, IRIS has been implemented in over 30 administrative localities in the UK. The trial and local evaluations of the IRIS implementation showed an increase in referrals from general practice to third sector DVA services with a variation in the referral rates within and across practices. Using Normalisation Process Theory (NPT), we aimed to understand the reasons for such variability by identifying factors that influenced the implementation of IRIS in the National Health Service (NHS). We conducted a mixed‐method process evaluation which included: (a) a case study (100 hr of participant observation, 19 interviews); (b) a survey (*n* = 118); (c) qualitative analysis of free‐text comments from the survey; (d) qualitative interviews (*n* = 8); (e) document review (*n* = 44). Data were collected from NHS and third sector staff across five London boroughs from August 2015 to December 2017, analysed descriptively and thematically and triangulated using the NPT constructs coherence, cognitive participation, collection action and reflexive monitoring. The survey showed wide variation in the extent to which practice staff saw IRIS as a normal part of their daily work. Qualitative data and documents illuminated drivers of DVA work, implementation barriers and suggested solutions. The drivers were related to individual professional's characteristics and relationships. The barriers were linked to the differing sense‐making and legitimisation of DVA work and differing contexts between the NHS and third sector. Solutions were adaptations to IRIS relative to these contextual differences. The suggested solutions can be used to update IRIS commissioning guidance, training for trainers and training for general practice. The updates should reflect the importance of ongoing support of IRIS from practice leads and commissioners, extended funding periods for IRIS and continuity of the IRIS team.

**What is known about this topic**

- IRIS is an intervention for general practice addressing domestic violence and abuse (DVA).
- After a trial showing increased rates of referrals from general practice to third sector domestic violence services, IRIS was widely implemented.
- As in the trial, local evaluations of the implementation found wide variation in referral rates within and across practices.

**What this paper adds**

- IRIS helped initiate and maintain the work of identifying and referring patients affected by DVA from general practice to the third sector.
- Variations in referral rates can be due to differing understanding of DVA among clinicians, which is influenced by both individual and practice level factors, with system‐level barriers acting as an additional challenge.

## 1 INTRODUCTION

Domestic violence and abuse (DVA) is a global public health and clinical problem (Department of Health, 2005; NICE, 2014; WHO, 2013) that causes significant morbidity and disability among women (Feder & Howarth, 2014). In the UK, the largest cost associated with DVA is to the National Health Service (NHS): £1.7 billion per year with the major cost borne by acute trusts and primary care (Walby & Olive, 2014). Almost all women with experience of DVA access the NHS regularly, either as the first or only point of contact with professionals (Department of Health, 2010). Although healthcare practitioners cannot meet all the needs of patients affected by DVA, they potentially play a pivotal role in the multisector response through identifying such patients and referring them to local DVA services (Garcia‐Moreno et al., 2015) which are largely based in the third sector.

The **I**dentification and **R**eferral to **I**mprove **S**afety (IRIS) intervention is a programme of training and support to improve the response to DVA in general practice. The programme focuses on the identification of women patients affected by DVA, an appropriate response by clinicians, and referral to a specialist, named IRIS advocate educator (AE) (Gregory et al., 2010) leading to increased safety and improvement in women's health and well‐being (Rivas et al., 2015). The IRIS model as intended is described in detail elsewhere (Sohal et al., 2018). Box summarises the implementation of the IRIS intervention as intended.

Box 1 **IRIS implementation as intended**

1 **I**dentification and **R**eferral to **I**mprove **S**afety (IRIS) integrates National Health Service (local health commissioners, general practices) and the third sector (local providers of domestic violence and abuse [DVA] services) in a multi‐sector response to DVA. A social enterprise IRIS Interventions (IRISi) supports the local commissioning, implementation, maintenance and growth of the IRIS programme (IRISi, 2017). IRISi managers raise awareness of the model across the UK and respond to interest from local health commissioners wanting to implement it. Commissioners appoint a third sector organisation (IRIS host) through a tendering process and identify a general practitioner (GP) interested in DVA to act as a clinical lead (CL). The host organisation recruits a specialist DVA worker. The GP and DVA worker receive training from IRISi in how to deliver the service and become the IRIS CL and IRIS advocate educator (AE) respectively. The commissioner covers the costs associated with these posts and pays an annual fee to IRISi. A local steering group of stakeholders is set up to monitor the implementation of IRIS. The CL and AE identify general practices interested in accessing the IRIS service and work with up to 25 practices to provide in‐house training, patient and professional resources and referral pathways for all patients affected by DVA. Resources include: (a) training pack; (b) referral forms; (c) care pathways; (d) electronic prompt in the medical record triggered by clinical presentation associated with DVA (Humiliate Afraid Rape Kick Safety [HARK] template); (e) DVA posters; (f) wallet size cards for patients. The AE is the named contact for patient referrals; she provides DVA advocacy to the patients, and DVA consultancy and ongoing support to practice staff. The practice is often used as a safe setting where the AE meets with referred patients, though meetings also happen within the community.

A pragmatic cluster‐randomised controlled trial showed that the IRIS intervention increased the rate of referrals to DVA services sevenfold (Feder et al.,^2011^). IRIS has also been found to be cost‐effective (Devine, Spencer, Eldridge, Norman, & Feder,^2012^) and acceptable to clinicians (Yeung, Chowdhury, Malpass, & Feder,^2012^) and patients (Malpass et al., 2014). Following the success of the trial, IRIS has been implemented in over 30 administrative localities in the UK. In line with the trial, local evaluations of IRIS implementation showed an increase in referrals from general practice to IRIS AEs with a variation in the referral rates within and across general practices (Howell, Johnson, Goddard, & Harrison, 2016; Johnson, Downes, Howell, Goddard, & Harrison, 2018). We hypothesised that the variability in the practice level outcome may reflect influential implementation factors. This study is aimed at understanding the reasons for the outcome variability by identifying factors that influenced the implementation of IRIS in the real‐world NHS.

## 2 METHODS

### 2.1 Design

Informed by the Medical Research Council guidance on evaluating complex interventions (Moore et al., 2015; MRC, 2008), we conducted a theory‐based mixed‐method process evaluation of the implementation of IRIS which included: (a) a case study; (b) a survey; (c) qualitative analysis of free‐text comments from the survey; (d) qualitative interviews; (e) document review. This process evaluation was carried out alongside the evaluation of the outcomes (Sohal et al., 2018) and cost‐effectiveness (Barbosa et al., 2018). The choice of the theoretical and analytical frameworks, study design and methods were influenced by the complexity of the IRIS intervention, the target audience of people involved in implementation of DVA programmes and the experience of the research team.

We conceptualised IRIS as a complex intervention (MRC, 2008) because the model: (a) includes several components; (b) requires changes in professional behaviour and ways of working at individual, organisation and inter‐organisation levels; (c) involves co‐ordinated work across NHS and third sector; (d) permits some adaptions to local context. The authors, with backgrounds in health services research (GF, CG, NL, AS), implementation science (CG, AD), health psychology (NL) and social science (AD), have approached the study with a paradigmatic perspective of critical realism (Shannon‐Baker, 2016).

This study was informed by Normalisation Process Theory (NPT) – a middle‐range socio‐behavioural theory (May & Finch, 2009) which has been most commonly used to assist understanding of interventions as part of feasibility studies and process evaluations (May et al., 2018). NPT offers a framework with four constructs and 16 sub‐constructs to assess the behaviour change and work that individuals and teams do to implement a new practice into their daily routine (Finch et al., 2013). In the context of IRIS, we conceptualised ‘practice’ (synonym ‘DVA work’) as the change in professionals' behaviour and ways of working leading to identification of patients with experience of DVA and referral to the IRIS service. We used the NPT framework to formulate propositions for the successful embedding of DVA work in the daily routine of general practice (see Table 1). We then interrogated our data against these propositions to identify implementation factors that could promote and inhibit their effectiveness and therefore explain the variation in referral rates within and across practices.

**Table 1:** Propositions for the successful embedding of DVA work in the daily routine of general practice mapped on the Normalisation Process Theory framework

NPT construct	Application to the normalisation of IRIS
1. Coherence – sense‐making work	DVA work should make sense to the general practice team and third sector organisation team (communal specification) and the individuals (individual specification); DVA work should match norms and values of NHS and third sector staff (internalisation); it should be distinct from other work and comprehensible to all the actors (differentiation).
2. Cognitive participation – relational work	NHS and third sector staff should work together to come to an agreement on DVA work (legitimisation); establish ways of working (enrolment); initiate DVA work with resources (initiation); and collectively establish ways to sustain it over time (activation).
3. Collective action – operational work in a given setting	NHS and third sector staff should have access to IRIS resources to support DVA work and use these resources in the context (contextual integration) and the group (relational integration); they should develop ways to work with each other and the resources to accomplish the DVA work (interactional workability) and figure out a way to divide labour to identify and care for patients with experience of DVA (skill‐set workability).
4. Reflexive monitoring – appraisal work	NHS and third sector staff should work out a system to define, collect and collate information about effects of IRIS (systematisation); work together and individually to appraise their DVA work and evaluate its worth (communal and individual appraisal); they should (if needed) modify IRIS for their context (reconfiguration).

Note

DVA: domestic violence and abuse; IRIS: Identification and Referral to Improve Safety; NHS: National Health Service; NPT: normalisation process theory.

**Figure 1:**
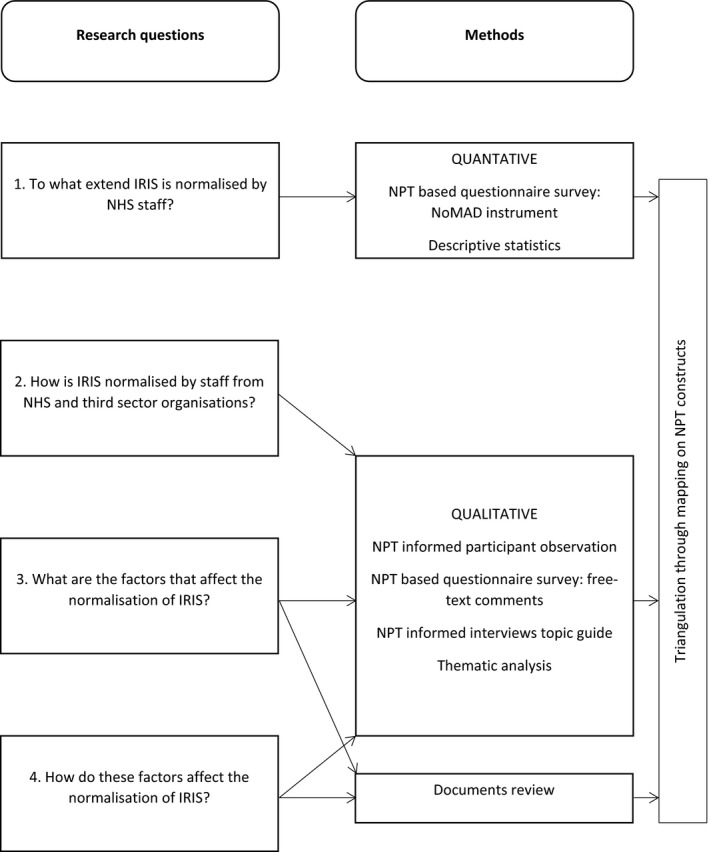
Research questions and methods applied in the process evaluation of the implementation of IRIS. NPT: Normalisation Process Theory

The choice of a mixed‐method approach was informed by prior research in the field of DVA (Bacchus, Buller, Ferrari, Brzank, & Feder, 2018; Hooker, Small, Humphreys, Hegarty, & Taft, 2015; Hooker, Small, & Taft, 2016) and was based on several assumptions. First, it allowed us to capture the complexity of the IRIS intervention and of the implementation context (Greene, 2007). Second, it helped to draw a more complete picture of the implementation process through answering different research questions (see Figure 1). Finally, it enabled the gathering of a wider range of data from multiple sources. This was important to compensate for poor engagement of general practice staff in research known from previous studies (Lewis et al., 2017; Parkinson et al., 2015). The use of NPT throughout the collection and analysis of quantitative and qualitative data helped to overcome some of the epistemological challenges of combining data (Farr et al., 2018). We followed a triangulation protocol as described by O'Cathain et  al (O'Cathain, Murphy, & Nicholl, 2010). Four data sets were collected separately and analysed using the NPT framework. The triangulation took place at the analysis and interpretation stage through mapping quantitative and qualitative results onto the NPT constructs.

### 2.2 Data collection

This study took place in five London boroughs (local government districts) which implemented IRIS between November 2010 (when the trial ended (Feder et al., 2011) and national commissioning began) and December 2017. The start date of IRIS implementation in each locality was defined as the date of the first IRIS training session delivered in a general practice within the borough. Study participants included a cross section of all those involved in the organisation and delivery of IRIS – AEs, clinical leads (CLs), local health commissioners and staff from general practices. Data collection took place between August 2015 and December 2017.

First, we carried out a case study in one locality. Then we undertook concurrent data collection across all localities using: (a) a survey with NHS participants; (b) qualitative interviews with NHS and third sector participants; and (c) a review of documents from NHS and third sector organisations. Findings from the case study informed the design of the survey and interview topic guides. This study was approved by the Research Ethics Committee at Queen Mary University of London (QMERC2015.29b 30.07.2015 and QMREC1799a 25.08.2016), local R&D (192654 29.02.16) and the Health Research Authority (16/HRA/4398 13.10.2016).

#### 2.2.1 Case study

During the first 12 months, an in‐depth ethnographic case study was initiated to develop and analyse data to test the concepts of NPT and their relevance to the wider study. This case study was selected based on it being an instrumental case (Stake, 1995), that is, an example of IRIS that was considered to be very similar to the model developed during the trial (Feder et al., 2011) and which could shed light on other cases. These data provided insights which informed the development of subsequent data collection procedures across the all wider study localities.

IRISi provided a shortlist of potential localities in the London area, excluding localities that had been involved in the original trial or delivered IRIS anomalously. One researcher (AD) approached three potential sites and one (locality III) consented to participate. The researcher was permitted to conduct participant observation (Spradley, 1980) and interviews (Rapley, 2004). Participant observation was undertaken to gather insight into the organisation of the IRIS (attending organisational delivery and steering group meetings) and the delivery of training (attending general practice IRIS training sessions). NHS interview participants were purposively sampled for diversity in career stage (early‐, mid‐ and senior) and familiarity with IRIS (referring/non‐referring).

#### 2.2.2 Survey

IRISi provided a list of all IRIS trained practices and their AEs from the five localities. The local Clinical Research Network sent an expression of interest email to practice managers in each practice on the list. Those interested in participating contacted the researcher (NL). She provided further information, obtained practice consent and sent the online survey link (Jisc, 2018) for forwarding to all practice staff, followed by two weekly reminders. The questionnaire included a socio‐demographic section, a standardised validated instrument (NoMAD) and space for free‐text comments. The NPT‐based instrument NoMAD (Finch et al., 2013, 2018; Rapley et al., 2018) measured individual's opinions on the levels of IRIS embeddedness in daily work. The NoMAD instrument consists of two sections: (a) three general questions about normalisation of the practice (a 10‐point scale from 0 ‘Not at all’ to 10 ‘Completely’) and (b) 23 items reflecting the four NPT constructs (Finch et al., 2015) (a 5‐point scale from 1 ‘Strongly disagree’ to 5 ‘Strongly agree’ with 0 for ‘Not relevant to me’). The 23‐item NoMAD instrument demonstrated good face validity, construct validity and internal consistency. We customised the original NoMAD instrument for the evaluation of IRIS (see Table 2). The customisation was informed by the case study and took place through consultations with instrument authors and IRIS providers, two pilots and three revisions.

**Table 2:** Responses to NoMAD instrument

NPT construct	NPT sub‐construct	NoMAD instrument item	*N*	Disagree, *n* (%)	Neither agree nor disagree, *n* (%)	Agree, *n* (%)	Not relevant to me, *n* (%)
1. Coherence	1.1. Differentiation Does DVA work differ from other practices?	I can see how identification of domestic violence and referral to the IRIS service differs from usual ways of working	108	12 (11)	37 (34)	50 (46)	9 (9)
	1.2. Communal specification Does DVA work make sense to the professional group?	Staff in my practice have a shared understanding of the purpose of the IRIS service	106	8 (8)	12 (11)	82 (77)	4 (4)
	1.3. Individual specification Does DVA work make sense to the individual?	I understand how being able to identify domestic violence and refer to the IRIS service affects the nature of my own work	108	7 (6)	18 (17)	74 (69)	9 (8)
	1.4. Internalisation Does DVA work link to personal norms and values?	I can see the potential value of the IRIS service for my work	108	1 (1)	9 (8)	92 (86)	6 (5)
2. Cognitive participation	2.1. Initiation Do implementers initiate DVA work in specific times and places, with resources?	There are key people in my practice who drive the IRIS service forward and get others involved	106	14 (13)	38 (36)	48 (45)	6 (6)
	2.2. Legitimation Do they work together to agree on the DVA work?	I believe that identifying domestic violence and referring to the IRIS service is a legitimate part of my role	107	0 (0)	7 (7)	91 (85)	9 (8)
	2.3. Enrolment Do they find ways to work together to engage in DVA work?	I'm open to working with colleagues in new ways to identify domestic violence and refer to the IRIS service	108	0 (0)	3 (3)	99 (91)	6 (6)
	2.4. Activation Do they collectively work out ways to sustain IRIS over time?	I will continue to identify patients with experience of domestic violence and refer them to the IRIS service	108	0 (0)	7 (7)	92 (85)	9 (8)
3. Collective action	3.1. Interactional workability Do implementers develop ways to work with each other and use resources to accomplish the DVA work?	I can easily integrate identification of domestic violence and referral to the IRIS service into my existing work	108	4 (4)	24 (22)	68 (63)	12 (11)
	3.2. Relational integration Do they build accountability and maintain confidence in the DVA work and each other?	Identifying domestic violence and referring to the IRIS service disrupts working relationships in my practice	108	84 (78)	11 (10)	3 (3)	10 (9)
		I have confidence in my colleagues' ability to identify domestic violence and refer to the IRIS service	108	1 (1)	11 (10)	91 (84)	5 (5)
	3.3. Skill set workability Have they divided the labour out and know who will do what and how to accomplish the DVA work?	Work of identification of domestic violence and referral to the IRIS service is assigned to those with appropriate skills	108	17 (16)	27 (25)	59 (55)	5 (4)
		Sufficient training is provided to enable staff to identify domestic violence and refer to the IRIS service	106	13 (12)	23 (22)	64 (60)	6 (6)
	3.4. Contextual integration Do they have access to adequate resources and use them to accomplish the DVA work?	Sufficient resources are available in my practice to support identification of domestic violence and referral to the IRIS service	107	9 (8)	20 (19)	72 (67)	6 (6)
		Practice management adequately supports identification of domestic violence and referral to the IRIS service	108	2 (2)	26 (24)	75 (69)	5 (5)
4. Reflexive monitoring	4.1. Systemisation Have implementers developed a system to evaluate the effect of their DVA work?	I am aware of feedback/reports about the effects of the IRIS service	107	41 (38)	18 (16)	36 (34)	12 (11)
	4.2. Communal appraisal Do they work together to evaluate the worth of their DVA work?	Practice staff agree that the IRIS service is worthwhile	107	2 (2)	17 (16)	82 (77)	6 (5)
	4.3. Individual appraisal Do they evaluate the effect of the DVA work on their own practice?	I value the effects that the IRIS service has had on my work	105	5 (5)	24 (23)	60 (57)	16 (15)
	4.4. Reconfiguration Do they do any work to redefine or modify their DVA work based on the evaluation?	Feedback from the IRIS service can be used to improve identification and referrals of patients with experience of domestic violence	108	3 (3)	17 (16)	80 (74)	8 (7)
		I can be flexible in how I identify domestic violence and refer to the IRIS service	108	0 (0)	22 (20)	72 (67)	14 (13)

Note

IRIS: Identification and Referral to Improve Safety; *N*: total number of responses to each NoMAD item; *n*: number of negative, neutral and positive responses to each NoMAD item.

#### 2.2.3 Qualitative interviews

Researchers (NL, AD) agreed to conduct up to 15 semi‐structured interviews (Murphy, Dingwall, Greatbatch, Parker, & Watson, 1999) with general practice staff and AEs, following the Malterud et al (Malterud, Siersma, & Guassora, 2015) approach to determining the ‘information power’ required to generate sufficient insight for the study. The interview topic guide, which was informed by previous research (Hooker et al., 2015) and our case study, reflected the four NPT constructs. We piloted and refined the topic guide during the first three interviews.

To reduce research burden, we recruited interview participants from the survey sample across four of five participating boroughs (except locality III previously involved in the case study). We drew a purposive sample of NHS and third sector participants in relation to locality, professional roles (AE, clinical and non‐clinical practice staff) and familiarity with IRIS (referrer/non‐referrer). First, we invited all IRIS AEs working with survey practices to participate in the study. Then we selected one survey practice in each locality with a midlevel rate of referrals to IRIS and asked their AE to send our interview invite to practice manager, referring clinician and non‐referring clinician. The researcher (NL, AD) obtained informed consent from professionals who expressed interest and arranged interviews at a convenient format, time and place. All interviews were audio recorded and transcribed. From the case study data set in locality III, we sampled four interviews transcripts meeting the above criteria.

#### 2.2.4 Document review

Researchers (NL, AS, AD) collected emails, meeting minutes, reports and other working documents from IRISi, NHS and third sector organisations in each locality (Shaw, Elston, & Abbott, 2006). Documents were included if they comprised implementation data relevant to the study period.

### 2.3 Analysis

#### 2.3.1 Quantitative data

Survey responses were downloaded from the Online Surveys platform (Jisc, 2018), cleaned and imported into Stata 15. Counts and frequencies were used to describe the sample and summarise NoMAD responses (Finch et al., 2018; Rapley et al., 2018). The customised 23‐item NoMAD instrument demonstrated a very good reliability of the whole scale (Cronbach *α* = 0.94) and the four NPT constructs (coherence *α* = 0.86, cognitive participation *α* = 0.86, collective action *α* = 0.80, reflexive monitoring *α* = 0.83) (DeVellis, 2012; Tavakol & Dennick, 2011). To simplify reporting, we collapsed the 10‐point and 5‐point scales to 3 points (1 ‘Not at all’, 2 ‘Somewhat’, 3 ‘Completely’ and 1 ‘Disagree’, 2 ‘Neither agree nor disagree’, 3 ‘Agree’ respectively).

#### 2.3.2 Qualitative data

Interview audio recordings were professionally typed using intelligent verbatim, checked against the original audiotape, anonymised and imported into NVivo 10. Free‐text survey comments were copied and pasted into a Word document and imported into NVivo 10. Researchers worked in parallel on the case study (AD) and mixed‐method data set (NL). We used both an inductive approach, informed by Glaser and Strauss (Glaser & Strauss, 1967), and a deductive approach using NPT as the analytical framework (Finch et al., 2015). First, we coded inductively discussing findings regularly to ensure reliability. Then, we organised codes into themes summarising influential implementation factors and grouped them under the NPT constructs and sub‐constructs. The NPT framework allowed us to frame the findings in the language of the theory and to provide a structure for combining quantitative and qualitative data.

#### 2.3.3 Documents review

Researchers (NL, AD) read and re‐read each document, extracted data on core implementation information into a Word table, then NL finalised the table.

## 3 FINDINGS

**Figure 2:**
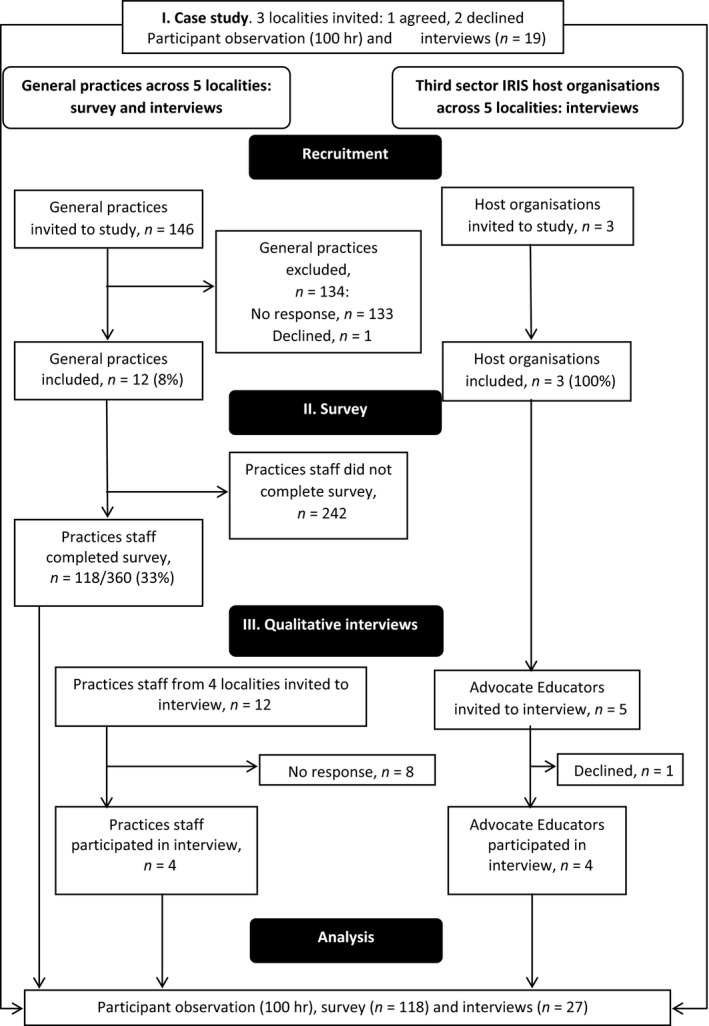
Flow of participants through the study

The flow of participants through the study is shown in Figure 2. At organisation and individual level, response rates were much higher within the third sector compared to the NHS. The final dataset included 100 hr of participant observation (case study), 118 survey responses, 27 qualitative interviews (19 from case study and eight from later phase) and 44 documents (see Data S1). Qualitative interviews (22 face‐to‐face, five telephone) lasted between 20 and 78 min, with a mean duration of 50 min.

The survey sample was overrepresented by experienced female clinicians, 60% of whom had attended IRIS training and nearly half had referred patients to IRIS service (see Table 3). The interview sample included six female AEs practising between 6 months and 4 years and six members of staff from general practices (one practice manager, one healthcare assistant and four general practitioners GPs) practising between 7 months and 21 years. Of six practice staff, four attended IRIS training and four referred patients to IRIS.

**Table 3:** Participants in online survey

Characteristic	*n/N*	%	Mean	*SD*
Female	97/111	87		
Clinical job	78/112	70		
Job experience, years	103		10.5	8.6
Attended IRIS training	66/110	60		
Referred patient to IRIS	54/111	49		

Note. IRIS: Identification and Referral to Improve Safety; *n*: number of respondents with each characteristic; *N*: total number of responses to each characteristic question.

The document review showed that IRIS was funded by varied local health commissioners (one NHS Clinical Commissioning Group (CCG), two local authorities, two joint funders) and hosted by three third sector organisations (see Table 4). Two host organisations were charities specialising in DVA, based on an explicitly feminist perspective and one was a charity for people affected by crime and traumatic events. IRIS funding time periods ranged between 1 and 3 years. In general, there were more continuity in CLs' posts compared to AEs, with most third sector organisations going through several changes of AEs. We identified gaps in the provision of IRIS within three of five localities, coinciding with gaps in funding and staffing in third sector organisations. The duration of these gaps was between 3 and 7 months. The case study in locality III identified diverging interests among IRIS implementers. Thus, the IRIS service was commissioned by the CCG, but the contract was managed by the local authority, meaning that there were multiple different interests in what IRIS might achieve. The CCG was interested to see changes in health outcomes, whereas the local authority prioritised reduction of DVA risk and connection with other DVA services.

**Table 4:** Context of IRIS implementation

Locality	Funded, date	Funder	Training for trainers, date	IRIS training session 1, date	IRIS team	IRIS staff turnover	Gaps in IRIS provision
I	November 2010	Local Authority	? 2011September 2016 February 2017	2011	Two AEs, CL, manager employed by third sector specialist DVA agency	Seven changes of AEs, same CL	None
II	Mid 2013	2013−2016 NHS CCG 2016‐present NHS CCG and Health Education England	August 2013 September 2016	15.11.2013	Two AEs, CL, manager employed by third sector specialist DVA agency	Same AEs, two changes of CL	None
III^a^	September 2013	2013‐present NHS CCG	May 2014 June 2017	14.03.2014	Two AEs, CL, manager employed by third sector specialist DVA agency	Three changes of AEs, same CL	July 2016–February 2017
IV	April 2014	2014‐present Local Authority	? 2014 November 2016 December 2017	02.10.2014	Two AEs, CL, manager employed by third sector generic organisation	Four changes of AEs, same CL	August–October 2016
V	September 2014	2014−2016 NHS CCG 2017‐present NHS CCG and Local Authority	December 2014 September 2016 February 2017	29.01.2015	Two AEs, CL, manager employed by third sector specialist DVA agency	One change of AEs, same CL	March–October 2017

Note

?: missing data; AE: advocate educator; CCG: Clinical Commissioning Group, a clinically led statutory NHS body responsible for the planning and commissioning of healthcare services for their local area; CL: clinical lead; DVA: domestic violence and abuse; IRIS: Identification and Referral to Improve Safety ; NHS: National Health Service.

a Case study locality.

The document review and interviews with IRIS commissioners and AEs confirmed that there was a variation in referral rates among practice staff and between practices. The survey indicated that despite IRIS being introduced to practices several years previously, NHS participants were uncertain about the service. The three general questions showed that practitioners' perceptions of the IRIS service being a part of their daily routine were evenly distributed between ‘Not at all’ and ‘Completely’. Most respondents (55/105 [52%]) were unfamiliar with IRIS versus 41 (39%) who felt familiar and nine (9%) who were in‐between. Only 50 of 106 (47%) participants thought that IRIS is currently a normal part of their work, while 41 (39%) had an opposite opinion and 15 (14%) were unsure. Most participants expressed hope that IRIS would become a normal part of their work (61/105 [59%]), 26 (25%) were pessimistic and 16 (16%) remained uncertain.

Despite IRIS being targeted at the whole practice team, between 4% and 15% of responses to the 23‐item NoMAD instrument indicated ‘Not relevant to me’ (see Table 2). Another notable trend was a comparatively high proportion of neutral answers (3%–34%) indicating professional uncertainty about the service. Free‐text comments provided possible explanations: no direct contact with patients (*n* = 7) and not aware of IRIS being implemented in the practice (*n* = 4).

Qualitative findings were organised into the three themes (drivers of DVA work, implementation barriers and suggested solutions) under the four core NPT constructs. The drivers were related to individual professional's characteristics and professional relationships. The barriers were linked to the differing sense‐making and legitimation of DVA work and differing implementation contexts between NHS and third sector. Solutions represented IRIS adaptations to these contextual differences. We report findings according to the NPT constructs.

### 3.1 Coherence (making sense of DVA work)

The survey showed that DVA work was meaningful for most individuals and teams across general practices, although less than a half could differentiate it from routine practice (see Table 2). This can indicate either unawareness of the IRIS service or its normalisation to the extent that the service becomes part of routine practice. Qualitative data showed that all AEs and most NHS staff differentiated IRIS from other DVA services through it offering a way to directly share the work of providing care to patients with experience of DVA.

Qualitative data showed that there were differences in how individuals and teams made sense of DVA work. While IRIS matched norms and values of all AEs and IRIS teams within third sector organisations, the individual appraisal of the value of DVA work among NHS staff varied, with some participants more strongly internalising the value of the work than others:

I suppose there's a big difference between GPs and how much overlap they see between social stuff and medical stuff. So there are some GPs who will happily write letters to the council to support housing applications and that kind of thing. And then others who are just like that's not my job; I'm not going to do it. So I think if you're more a kind of biomedical person, like, I'm here to treat the illness that the patient has, then you're less wanting to get involved in social things. (GP01)

The collective specification of the purpose and nature of DVA work differed between NHS and third sector teams. Two AEs who came from third sector organisations with a feminist perspective acknowledged systemic differences between theirs and some clinicians' understanding of DVA and attitudes towards abused women. They thought that these individual beliefs and attitudes did not align with the feminist conceptualisation of DVA within IRIS and that such conflict could partly explain poor engagement of some general practice staff in DVA work:

…I think it's also they don't understand domestic violence. I delivered a talk to the PLT [Protected Learning Time] for GPs, and it just gives you an idea – I had an Asian woman doctor, and she just happened to be Asian and she was a woman doctor, and we were talking about if they disclose, you believe. You believe. And she went, “But what if she provoked it?” And I said, “But how can you provoke violence? How could you do that?” And then she just shuffled, and she said, “Well, sometimes it's an argument.” I said, “No, if it's an argument, that's not an issue. An argument is healthy on occasions and there's no fear involved, and it shouldn't result in abuse. If it does, then that is abuse, isn't it?” And then I had somebody else say to me, when I said about disclosure, and they said, “What if she's lying?” So some have already got these barriers, haven't they, and you know the ones that just do not get it – do not get the whole thing about domestic violence – so you know that they will never refer to you. (AE01)

### 3.2 Cognitive participation (enrolling people into DVA work)

Most general practice staff were positive about participating in training and referring to IRIS, although less than 50% could identify DVA leads in their practice (see Table 2). The case study also found that while it was possible to initiate a new approach to DVA work through training, a barrier was having key people within practices supporting the ongoing activation of the work among the other demands of primary care environments.

Interviews and documents showed that AEs were the main driver for DVA work. They played an important role in bridging general practice and the third sector through training and enrolling NHS staff into DVA work, helping to reorganise the work of responding to DVA by taking on the responsibility of providing DVA consultancy to practice staff and DVA advocacy to patients. Barriers to this were differing attitudes within general practice towards the legitimacy of DVA work, with AEs sometimes struggling to change beliefs about DVA and the role of the GP in addressing it.

Most NHS and third sector participants highlighted systemic contextual barriers to IRIS uptake (e.g. increasing demand on general practice, competing priorities):

I think the barriers are there, them not having, GPs not having necessarily enough time, I know they have their hours a week or whatever dedicated but when there's so many things coming up and so many changes and all this, I think sometimes if they, it [IRIS] can be just seen as slightly as an after, oh that's a really nice add on but we need to get the, I don't know, the diabetes training done first because that's sort of a requirement that's come in.” (AE03)

Two AEs suggested increasing the uptake of the service through including IRIS training as a module of the mandatory safeguarding training.

While all AEs valued the flexibility of the training in making it relevant to the local context, most NHS and third sector participants identified two contextual barriers to training reach: logistical challenges of arranging training for the whole practice team and staff turnover. Participants saw the opportunity to increase the reach through further adaptations to the practice context – update IRIS training to fit into the overstretched general practice and provide additional training for new staff.

### 3.3 Collective action (enacting DVA work within context)

Most respondents reported that they have received sufficient training and resources for enacting DVA work within their practice, although up to 25% were not sure if this work was assigned to those with appropriate skills (see Table 2). In line with the survey, most interview participants acknowledged the importance of the IRIS training and peer influence in initiating DVA work. However, differing organisational cultures of general practice and the third sector made it challenging to enact IRIS and sustain it over time. AEs had to undertake a long period of invisible work establishing relationships and building trust to get into general practices before the intervention could begin. This demanded significant flexibility on the part of the service, as these periods of invisible work did not result in any referrals. After the service was launched, AEs used constant reminders and visits to practice to maintain the rates of referrals.

While there was confidence in the allocation of work between general practice and third sector organisations, short‐term funding for the IRIS host organisation, resulting in professional uncertainty and staff turnover, made it difficult for participants to build confidence in one another and embed DVA work into their routine practice:

And that's what GPs have said, like that's what I've heard a lot of practitioners say, is that, “What we find that it's funded for a year and then the service is no longer there.” So that's not building that confidence for them as well, that relationship then, building that long term relationship, you know, in the primary care sector because if these type of service are just disappear I think, they don't have anyone to refer to, you know, they're not going to build those relationships. (AE04)

The decommissioning and recommissioning of the service is a real hassle on the front line. (FTGP1)

Further, half of AEs felt that NHS culture and processes were too slow and bureaucratic to match the nature of the DVA work which they conceptualised as an emergency:

The CCG have been very … they're not being obstructive ‐ they just go at their pace as though they have all the time in the world. They're very demanding when they want information, but they're not as forthcoming when you need it, so I find that can be difficult. (AE01)

The overall emotional toll of general practice was mentioned as one of the factors preventing some clinicians from consistently asking about DVA:

Some of the GPs may just be generally exhausted and tired from the demands of their role and they just want to stick to the basic requirements, which is, ‘I have eight minutes clinical time to see a patient, to assess, diagnose, treat; two minutes for my notes, and that's all I'm prepared to do.’ (AE02)

Although most participants valued IRIS' physical resources (referral pathways, DVA posters, patient cards, HARK template), some were unsatisfied due to the inconsistent supplies of posters and cards and technical limitations of the HARK template (repeated pop‐ups even after DVA has been recorded, inability to analyse template usage for appraisal purposes).

### 3.4 Reflexive monitoring (monitoring and sustaining DVA work over time)

The survey showed that most NHS staff felt that IRIS is worthwhile and IRIS feedback can be used to improve the service. However, only a third of respondents were aware of any IRIS reports and only half could assess the effect of IRIS on their own practice (see Table 2). Qualitative data also showed that the feedback component of the IRIS model was not implemented as intended. NHS staff and third sector staff had conflicting perspectives on the consistency of feedback about IRIS. While there were formal systems of collecting and reporting information on the progress of IRIS, and strong individual appraisal of the service as successful on the part of third sector organisations and funders, communal appraisal of the work was not being achieved through the current system of formal feedback to general practices. Interestingly, good performance on all agreed metrics for monitoring did not protect the service from a period without funding in three of five localities (see Table 4).

According to AEs, monitoring made up a large part of their workload, which they felt took away from the time they could spend engaging with practice staff and patients. In contrast, most clinicians reported receiving useful feedback on the IRIS service from their patients, and few acknowledged receiving it from their AEs. This discrepancy can be explained by the differences in perceptions of satisfactory communal appraisal between clinicians and AEs. Thus, the former wanted feedback not only on whether the patient they referred had received the service but on what a ‘good outcome’ might look like for that patient. This could be argued to link to the construct of coherence, where there was limited communal specification of DVA. This in turn may make it challenging to establish a shared vision of what improvement entails. Informal channels of communication were more successful at building a shared sense of progress between clinicians, AEs and patients:

Certain GPs who refer, well I'd say their referrals increased but the ones who have referred and maybe we've given really diligent feedback to, or perhaps had to call them a couple of times or provided them with additional information, then they've been the ones who have really, the referrals just keep coming and it seems to be less about, okay we've trained you now so now the referral comes in but actually they really liked how we helped their previous client, they think this is a great idea. (AE03)

Suggested areas for improvement covered a combination of informal individual and formal communal appraisal. Thus, clinicians wanted annual formal feedback from IRIS showing how each practice performed compared to the rest of the borough.

## 4 DISCUSSION

This study offers detail and insight about IRIS implementation in the real‐world NHS, demonstrates the benefit of a mixed‐method process evaluation for complex intervention implementation, and adds to knowledge about the use of NPT in process evaluations. We found that IRIS successfully facilitated the shared work of providing whole‐person care to patients with experience of DVA through linking healthcare in general practice and DVA advocacy in the third sector. All third sector participants and most NHS staff showed high individual specification of DVA work, although the latter demonstrated varied legitimisation of the DVA work. The collective specification between NHS and third sector teams was less strong due to the differences in organisations' ethos and culture. The AE emerged as a critical component of IRIS implementation, acting as a broker between the differing organisational cultures of primary care and third sector (Long, Cunningham, & Braithwaite, 2013). She initiated and activated the service through establishing trusting relationships with general practices and maintaining confidence in the service. IRIS activation was supported by the continuity of AE contact and adaptability of the service to the demands of general practice. Differing cultures between NHS and the third sector, high demand and competing priorities within general practice, lack of support from commissioners and practice leadership and logistical problems with arranging IRIS training and supplying IRIS physical resources challenged IRIS initiation, activation and contextual integration. Another barrier was the short‐term funding of IRIS resulting in staff turnover in the host organisation, professional uncertainty and loss of trust in service across both sectors. Although NHS and third sector participants agreed on the importance of reflexive monitoring in sustaining their DVA work, the teams had conflicting views on what a satisfying feedback component of IRIS should look like. Contextual integration can be improved through further adaptations of IRIS to the NHS context. Reflexive monitoring can be enhanced through aligning clinicians' and AEs' understanding of a positive outcome of the IRIS intervention and combining informal and formal feedback to general practices.

We hypothesised that centralised training for IRIS teams and provision of IRIS training and ongoing support by the same AE and CL to all the practices in a locality indicates high consistency of the intervention on the part of the third sector organisation. Our data suggest that the flexibility of the AE in their relationship‐building with general practice was a key contributing factor to overall programme consistency. This speaks to the notion of intervention ‘plasticity’ that May et al (May, Johnson, & Finch, 2016) have described as being important for successful implementation.

Our findings on the implementation barriers offer an explanation for why local evaluations of the IRIS implementation, similarly to the original trial, found variation in the referral rates within and across general practices. Differing understanding of DVA work among clinicians, which was influenced by both individual and practice level factors, with system‐level constraints acting as an additional challenge, can contribute towards varied referral rates despite the hypothesised high consistency of the intervention on the part of the third sector host organisation.

Our findings on a strong sense of coherence of DVA work with individual healthcare professionals' roles and on system‐level barriers to initiating DVA work are in line with process evaluations of DVA interventions in the trial context (Yeung et al., 2012; Hooker et al., 2015). Barriers to inter‐agency collaboration in the provision of whole‐person care to patients affected by DVA including systemic differences in collective specification, legitimisation and contextual integration have been identified in previous research (Garcia‐Moreno et al., 2015; Szilassy et al., 2016), although only one study evaluated a post‐trial implementation of a DVA intervention in healthcare (Hooker et al., 2016). The authors found barriers to the sustainability of the DVA programme in the same domains as in our study – coherence, contextual integration and reflexive monitoring.

### 4.1 Strengths and limitations

The strengths of this process evaluation include a mixed‐method approach and theory‐informed analysis and interpretation of findings. Quantitative tools included a reliable validated NoMAD instrument. Qualitative study with NHS and third sector participants gave equal voices to both professional groups and illuminated converging and conflicting perspectives within and between the groups. Involvement of researchers with multidisciplinary backgrounds throughout data analysis broadened the interpretation of findings.

Study limitations are related to poor engagement of general practice staff in the research, which has been previously reported (Lewis et al., 2017; Parkinson et al., 2015). Previous research found that recruitment of general practitioners in studies was inhibited by the personal and professional factors (e.g. no interest in research, need to prioritise clinical care over research, lack of protected time for research) (Sahin, Yaffe, Sussman, & McCusker, 2014). A low response rate to the survey and qualitative interviews limited the generalisability of the quantitative results and transferability of the qualitative findings. It is possible that our participants were more likely to have positive views of IRIS. Therefore, our findings could be positively skewed, and we may not have captured all implementation barriers. Finally, the absence of patients' perspectives does not allow to draw a full picture of all the factors that could have had an impact on the IRIS implementation.

### 4.2 Practical implications

We demonstrated how the mixed‐method approach and NPT framework can be used in the evaluation of a post‐trial implementation of a complex intervention across healthcare and third sectors. Our findings are relevant to the implementation and sustainability of any complex intervention which involves multi‐agency work when providing whole‐person care to patients with medico‐social problems. Solutions to implementation barriers can be used to update IRIS commissioning guidance (Howell & Johnson, 2011), IRISi training for trainers and IRIS training for general practices, and could be of relevance more broadly for DVA interventions in healthcare. Updates should reflect the importance of leadership with regard to DVA both within individual practices and by commissioning bodies, and the vital role of effectively managed communication between NHS and third sector practitioners for building shared understanding of the service. The damage to confidence in the service that results from the uncertainty of short‐term funding should also be emphasised. Updates could consider how IRIS training could be locally adapted to fit into an over‐burdened general practice, perhaps by blending face‐to‐face training with e‐learning.

### 4.3 Conclusion

The IRIS model facilitates behaviour change among general practice staff and collaboration between the NHS and third sector, with the aim of initiating and sustaining DVA work. The IRIS AE is the main driver of the IRIS model bridging the NHS and third sector, maintaining consistency of the core model components whilst adapting its delivery to fit into differing organisational contexts. Ongoing organisation and system‐level support from the funder and practice leadership enable DVA work to be sustained. Repeated training and the physical presence of the AE in the practice supports sustainability of that work. Continuous iterative evaluation and feedback acceptable to both NHS and third sector staff could improve appraisal of the DVA work. The approach taken in this paper demonstrates the value of conducting a theoretically informed process evaluation to further understanding of the implementation of complex interventions in real‐world settings.

## CONFLICT OF INTEREST

Natalia V. Lewis, Anna Dowrick, Alex Sohal, Chris Griffiths have no known conflicts of interest. Gene Feder serves on the IRISi advisory board.
