# Supplementary material for: Implementation of the Identification and Referral to Improve Safety programme for patients with experience of domestic violence and abuse: A theory‐based mixed‐method process evaluation
Source: Health Soc Care Community. 2019 Mar 13;27(4):e298–312. doi: 10.1111/hsc.12733 (PMC6617800; doi:10.1111/hsc.12733)
Supplement: Supplementary file 1 [file HSC-27-e298-s001.docx]

# Supporting material

## Items included in documents review

### From IRISi

IRIS national reports 2013, 2014, 2015, 2016

Email on training for trainers’ dates (15.01.2018)

Email on core implementation dates for five localities (15.12.2017)

### From locality I

Local area summary for IRIS national annual report Dec 2015

Domestic Violence and Abuse - Initial Needs Assessments 2015, 2017

IRIS Programme – Updates for Mental Health CCG Programme Board 2015, 2017

Identification and Referral to Improve Safety (IRIS) Service business case 2016

Contract award for Identification and Referral to Improve Safety (IRIS) 2016

IRIS service specification 2017

### From locality II

Local area summary for IRIS national annual report Dec 2015

DVA project steering group meeting reports Nov 2015, Jan 2016, May 2016, Dec 2016

DVA project steering group meeting minutes Nov 2015, March 2016, June 2016, Nov 2016, Mar 2017, June 2017, July 2017, Dec 2017

### From locality III

Steering group meeting reports Feb 2015, May 2015, July 2015, Sept 2015, Nov 2015, June 2016

IRIS business case Feb 2016

IRIS Welcome Pack 2015

Joint Health and Wellbeing Strategy 2016-18: Living well, working together (2016)

### From locality IV

Local area summary for IRIS national annual report Nov 2015

IRIS annual report 2015-2016

Steering group meeting report Jan 2016

Email from AE (05.08.2016)

### From locality V

CCG Annual report and accounts 2013-2014, 2014-2015, 2015-2016

Local area summary for IRIS national annual report Nov 2015
